# Empowering entity synonym set generation using flexible perceptual field and multi-layer contextual information

**DOI:** 10.1371/journal.pone.0321381

**Published:** 2025-04-21

**Authors:** Subin Huang, Daoyu Li, Chengzhen Yu, Junjie Chen, Qing Zhou, Sanmin Liu

**Affiliations:** School of Computer and Information, Anhui Polytechnic University, Wuhu, Anhui, China; Sreenidhi Institute of Science and Technology, INDIA

## Abstract

Automatic generation of entity synonyms plays a pivotal role in various natural language processing applications, such as search engines, question-answering systems, and taxonomy construction. Previous research on generating entity synonym sets has typically relied on approaches that involve sorting and pruning candidate entities or solving the problem in a two-stage manner (i.e., initially identifying pairs of synonyms and subsequently aggregating them into sets). Nevertheless, these approaches tend to disregard global entity information and are susceptible to error propagation issues. This paper introduces an innovative approach to generating entity synonym sets that leverages a flexible perception mechanism and multi-layer contextual information. Firstly, to determine whether to incorporate new candidate entities into synonym sets, the approach integrates a neural network classifier with a flexible perceptual field. Within the classifier, the approach builds a three-layer interactive network, and connects the entity layer, set layer, and sentence layer to the same embedding space to extract synonym features. Secondly, we introduce a dynamic-weight-based algorithm for synthesizing entity synonym sets, leveraging a neural network classifier trained to generate entity synonym sets from the candidate entity vocabulary. Finally, extensive experimental results on three public datasets demonstrate that our approach outperforms other comparable approaches in generating entity synonym sets.

## Introduction

An entity synonym set consists of names or terms that convey similar or identical meanings. For instance, {“USA”, “U.S.”, “United States”} is a synonym set, where each term refers to the same nation [1–3]. The identification of entity synonym sets has become a crucial undertaking, offering substantial advantages for various downstream applications, including search engines [4–6], question answering systems [7–9], and taxonomy construction [10–12]. For example, when analyzing the query “The capital of the U.S. is D.C.”, an intelligent system must accurately interpret “U.S.” as “United States” and “D.C.” as “Washington.” This understanding is critical for satisfying the informational demands of users [13].

Currently, most research on generating entity synonym sets falls into two main categories: ranking and pruning approaches [14–16] and two-stage methods [17–19].

- **Ranking and pruning approaches.** Candidate terms are ranked according to their probability of referring to the same entity, and the ranked terms are then pruned into a set of entity synonyms. By treating each term in the glossary as a query, the approach ultimately outputs a complete set of entity synonyms derived from the glossary.
- **Two-stage task approaches.** These approaches divide the process of generating synonym sets into two cohesive steps. Initially, a synonym detection technique is employed to identify candidate synonym pairs of terms. Subsequently, a synonym set generation algorithm is utilized to combine these pairs to form a synonym set.

However, the above solution, while valid, suffers from the following two drawbacks:

- **Ignoring the global information of candidate entities.** The ranking and pruning approach employs a rank-and-prune strategy, which first ranks candidate terms according to their probability of referring to the same entity. Subsequently, the sorted list is pruned to create entity synonym sets. However, these approaches handle each candidate separately and independently calculate the probability of its reference to the queried entity, thereby overlooking the global information shared among candidate entities. Essentially, harnessing such global information could significantly enhance the quality of synonym set discovery.
- **Accumulation of error propagation.** The two-stage approach include the initial extraction of entity synonym pairs, which are then combined into entity synonym sets. However, these approaches rely solely on the training data in the first stage and fail to fully utilize training signals in the second stage. Furthermore, during the organization of entity synonym sets, the identified entity synonym pairs are usually fixed, lacking a feedback mechanism between the first and second stages. This absence of feedback can result in the accumulation of errors.

This paper proposes a framework named EnSynFields to overcome the previously mentioned limitations in generating entity synonym sets. EnSynFields improves the extraction of sentence-layer global information for entity synonym sets, providing flexible field awareness for their automated generation. Specifically, EnSynFields consists of the following main components:

- **Three-layer interaction field network.** We build a three-layer interaction field network, encompassing the entity layer, set layer, and sentence layer, to capture the interaction of global information among the candidate entities. A bidirectional propagation mechanism is proposed to learn entity feature representations across the three-layer interaction fields, thereby helping to alleviate the potential noise caused by relying on a single network layer.
- **Flexible perceptual field neural network classifier.** We build a flexible perceptual field neural network classifier to determine whether to incorporate new candidate entities into synonym sets. This classifier jointly models entities, sets, and sentences, learning to represent entity synonym sets based on the three-layer interaction field.
- **Dynamic-weight-based set generation algorithm.** We propose a dynamic-weight-based set generation algorithm for generating entity synonym sets. This algorithm utilizes a weighted cross-entropy loss function to balance the distribution of samples among the generated sets, thereby mitigating error propagation stemming from the flexible perceptual field neural network classifier.

This study makes the following contributions:

- By considering multi-layer contextual information, our approach jointly learns the representations of entities and synonym sets within a network learning framework. This is achieved by encoding contextual information at three levels: the entity layer, set layer, and sentence layer.
- A flexible perceptual field neural network classifier is proposed for the holistic modeling of entity synonym sets. Integrated with a dynamic-weight-based set generation algorithm, it effectively constructs new synonym sets.
- Numerous experiments were conducted on three real-world datasets to assess the efficacy of our proposed EnSynFields framework. Experimental results demonstrate that our approach outperforms existing methods, validating its effectiveness.

In the subsequent sections, this paper is structured as follows: The “Related Work” section provides a concise overview of existing techniques for synonym set generation and highlights the key features of our proposed approach. The “Methodology” section details our proposed framework. The “Experiments” section presents experimental results and an in-depth analysis. Finally, the “Conclusion” section summarizes the key findings of this study and outlines future research directions.

## Related work

This section explores two correlated approaches in the generation of entity synonym sets: the ranking and pruning techniques, and the two-stage task strategies.

### Ranking and pruning approaches

Ranking and pruning approaches involve associating a given query term with an entity. Initially, candidate terms are evaluated based on their likelihood of denoting the same entity, followed by pruning the ranked list to generate the final synonym set. Viewing each vocabulary term as a query, these approaches ultimately result in synonym sets for all entities in the vocabulary [13].

Ranking-based approaches aim to improve the quality of generated synonyms by ranking them according to their relevance to the original word. Qu *et al*. [20] introduced a machine learning-based sorting framework to rank generated synonyms by learning weights, thereby improving their relevance. In addition, Yu *et al*. [21] proposed a corpus-based ranking approach that leverages a large-scale corpus to learn the distribution of synonyms for more accurate ranking.

The goal of the pruning technique is to eliminate low-quality or irrelevant candidates from the generated synonyms. Zhang *et al*. [22] developed a pruning approach based on syntactic and semantic information to filter out non-contextualized synonyms, highlighting the significance of pruning in synonym generation. In addition, Qu *et al*. [23] proposed a pruning strategy based on an attention mechanism, where the model automatically focuses on the most relevant synonyms, thereby reducing redundancy and irrelevant words in the generated set.

### Two-stage task approaches

Two-stage task approaches implement a pair of sequential subtasks to discern sets of synonyms for entities. Initially, they focus on developing a model for synonym prediction, which evaluates the synonymous nature of proposed pairs of strings. Subsequently, these approaches utilize an algorithm for synonym expansion in conjunction with the previously mentioned model to generate synonym sets. Typically, these two-stage task approaches excel in discerning semantic connections among candidate strings, thereby efficiently collating synonymous terms [2, 13].

Ren and Cheng [17] developed an approach that combines a heterogeneous graph-based data model with a ranking algorithm to discover synonyms in web text. Their approach incorporates string names, key structured attributes, subqueries, and web page connections to compile an expanded set of synonyms. Shen *et al*. [18] developed a technique that combines context-based feature selection with a ranking-based model for extracting synonym sets from text corpora. In a separate study, Shen *et al*. [13] introduced an approach for creating entity synonym sets, designing a classifier to validate string pair synonymy and employing an expansion algorithm to refine these sets.

Huang *et al*. [19] introduced an approach for forming Chinese entity synonym sets, comprising both extraction and refinement stages. In the initial phase, they employed a combination of direct, pattern-based, and neural network mining techniques to gather potential Chinese entity synonyms. In the subsequent refinement phase, they applied semantic rules, coupled with field-specific and similarity-based filtering, to enhance the accuracy of the extracted Chinese entity synonym sets.

### Related work discussion

Previously, we examined two approaches for synonym set discovery. This section provides an in-depth discussion of the salient aspects of our proposed approach and revisits the approaches previously addressed. The ranking and pruning approach can improve the quality of the generated set of synonyms; however, an overly aggressive ranking and pruning process may lead to the erroneous exclusion of some useful synonyms, thereby reducing the coverage of the synonym set. In addition, approaches that rely excessively on statistical and corpus information may not perform well when dealing with field-specific or low-resource languages, as such information may be insufficiently detailed. Most two-stage approaches are supervised, requiring a labeled dataset of synonymous words. Nevertheless, the availability of labeled synonym datasets is not consistently assured, and the development of such datasets can be a costly endeavor. This limitation hinders the scalability and broader applicability of these approaches.

This paper introduces an approach for the efficient generation of entity synonym sets, leveraging a flexible perceptual field and layered contextual insights. A three-layer interaction field network is constructed to learn entity representations in a mutually reinforcing manner, mitigating the noise introduced by rich contextual information. Specifically, to determine whether to include new candidate entities in synonym sets, the approach incorporates a neural network classifier with a flexible perceptual field. In the classifier, a three-layer interaction field network is employed to link entities, entity synonym sets, and entity sentences, encoding high-order context from a flexible perceptual field to extract synonym features. To overcome the previously identified constraints, this study presents an advanced dynamic-weight-based algorithm for generating synonym sets, utilizing a finely trained neural network classifier to extract these sets from a selection of candidate entities. Thorough experiments conducted on a variety of real-world datasets demonstrate the superiority of our approach over similar methods, confirming its effectiveness.

## Methodology

This section introduces key concepts related to our research and outlines the structure of our proposed approach. Additionally, it provides an in-depth analysis of this structure, exploring each of its components in detail.

### Problem formulation

**Entity.** Any distinct thing, object, or concept that exists independently or can be considered separately in reality. It may appear as either a single word or a phrase.

**Entity synonym.** A term (i.e., word or phrase) that has the same semantic meaning as another term in the same text corpus but differs in surface form.

**Set of entity synonyms.** A set containing entity names that either convey similar meanings or refer to an identical entity.

**Knowledge base.** A knowledge base is a collection of entities encompassing numerous facts. This paper specifically focuses on a particular type of fact: entity synonyms. These synonyms serve as initial seeds for discovering previously unknown synonym terms.

**Set-layer context.** A set-layer context is a semantic association within an interaction field network. If entity mention *M* links to a synonym set in this network, the synonym set is deemed the set-layer context of entity mention *M*. Similarly, a link between one synonym set $S_{1}$ and another synonym set $S_{2}$ within this network designates the latter as the set-layer context of the former.

**Flexible perceptual fields.** Given a text corpus *C*, flexible perceptual fields are sets of top-*k* sentences with the highest relevance scores from *C*. The relevance scores are computed based on entity perceptual fields within the interaction field network, utilizing the BM25 (Best Matching 25) information retrieval approach.

**Problem Definition.** Given a text corpus *T*, a knowledge base *K*, and a vocabulary *V* (a list of candidate entities) generated from *T*, our task aims to extend entity synonyms from *T* and *V* based on flexible perceptual fields and multiple layers of contextual information.

### Overview of framework

**Fig 1:**
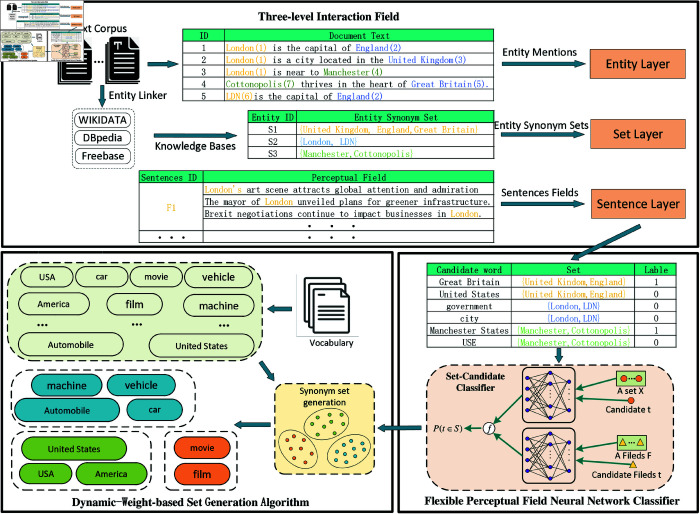
EnSynFields framework overview.

As shown in Fig 1, the EnSynFields framework comprises three key components: a three-layer interaction field network, a flexible perceptual field neural network classifier, and a dynamic-weight-based set generation algorithm.

1. **Three-layer interaction field network:** An interactive field is constructed to capture global information among candidate entities. The interaction field network includes contextual information from the entity layer, set layer, and sentence layer, helping to mitigate potential noise arising from individual fields.
2. **Flexible perceptual field neural network classifier:** This classifier is developed to assess whether new candidate entities should be incorporated into the current entity set. It features a network with a flexible perceptual field, enabling the learning of additional contextual characteristics.
3. **Dynamic-weight-based set generation algorithm:** A dynamic-weight-based algorithm for expanding entity synonym sets is devised. It resamples generated data and employs a weighted cross-entropy loss function to balance the distribution across various sets, thereby improving the efficiency of entity synonym set expansion.

### Building a three-layer interaction field network

**Fig 2:**
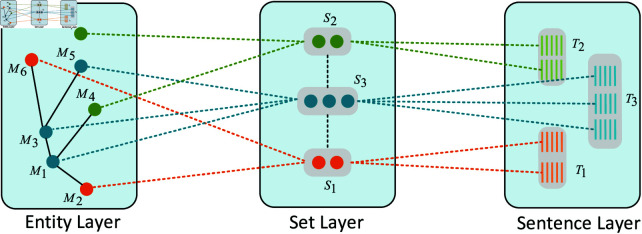
Three-layer interactive field network architecture.

As depicted in Fig 2, the three-layer interaction field network N{f(M−M), f(S−S), f(T−T)} consists of the entity-layer field *f*(*M*–*M*), set-layer field *f*(*S*–*S*), and sentence-layer field *f*(*T*–*T*). The three-layer interaction field network preserves contextual information at the entity layer, set layer, and sentence layer, offering flexible receptive fields for additional encoding of entities, sets, and sentences.

#### Data preparation.

To build a synonym set generative model, we begin with data preparation, focusing on collecting training and validation data. We collected the data using the following approach.

- **Entity data acquisition:** The existing named entity recognition tool [24] is used to identify entities mentioned in the text corpus, i.e., entity references.
- **Entity set data acquisition:** An established entity linker, such as DBpedia Spotlight [25], is utilized to associate entity references in the corpus with corresponding entities in the knowledge base. The set of synonym seeds from linked corpora serves as a distant supervision signal, aiding in the discovery of additional sets of entity synonyms from raw text corpora that are not present in the knowledge base.
- **Sentence set data acquisition:** The BM25 information retrieval algorithm [26] is employed to retrieve the five most relevant sentences from the original text corpus. This number was chosen as it balances providing sufficient context with maintaining computational efficiency. These selected sentences are scored based on factors such as word frequency, sentence length, and sentence frequency, and subsequently utilized as the sentence set for entities.

#### Building entity-layer field network.

**Fig 3:**
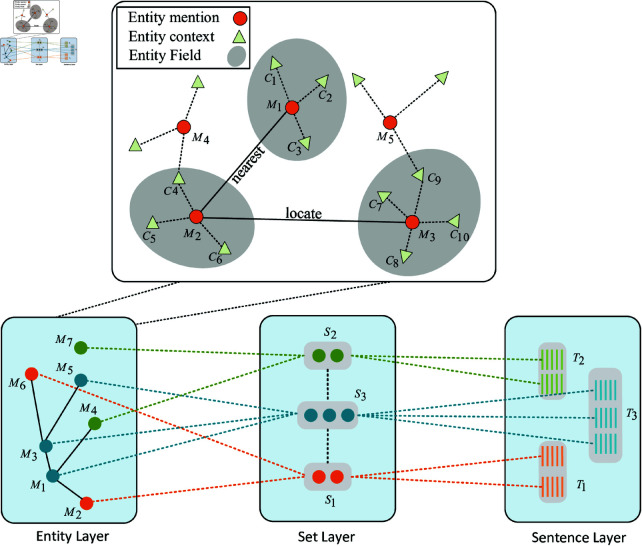
Entity-layer field network.

The goal of building an entity-layer field network is to establish entity-to-entity semantic relationships. As shown in Fig 3, the entity mentions *M* serve as the nodes of the network. To capture entity-to-entity semantic relationships, we construct the entity-to-entity semantic field network *f*(*M*–*M*) using the following rules:

- **Rule 1:** If $M_{i}$ is the nearest entity mention to $M_{j}$ in the text corpus, then we construct the network $f(M_{i}$ − $M_{j})$ using the top-*k* contextual information of entities $M_{i}$ and $M_{j}$. For example, the network of $M_{1}$ and $M_{2}$ is $f(M_{1}$ − $M_{2})=\{c_{1},c_{2},c_{3},c_{4},c_{5},c_{6}\}$.
- **Rule 2:** If $M_{i}$ and $M_{j}$ appear in the same sentence in the text corpus, then we construct the network $f(M_{i}$ − $M_{j})$ using the top-*k* contextual information of entities $M_{i}$ and $M_{j}$. For example, the network of $M_{2}$ and $M_{3}$ is $f(M_{2}$ − $M_{3})=\{c_{4},c_{5},c_{6},c_{7},c_{8},c_{9},c_{10}\}$.

The entity-layer field network implicitly encodes semantic information among entities, facilitating the comprehensive capture of semantic relationships within this specific layer.

#### Building set-layer field network.

**Fig 4:**
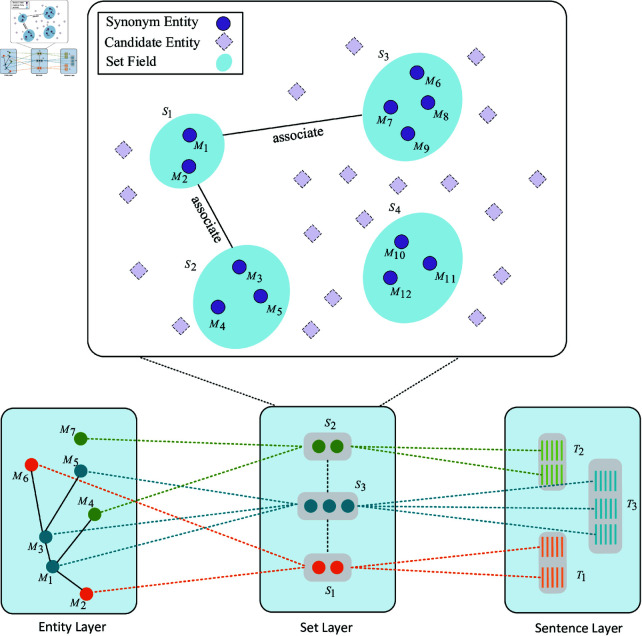
Set-layer field network.

We construct a set-layer field network to capture set-layer contextual information. As illustrated in Fig 4, the set-layer field network consists of numerous candidate entities. To capture set-to-set semantic relationships, we construct the set-to-set semantic field network *f*(*S*–*S*) using the following steps:

- First, if the entity mentions $M_{i}$ and $M_{j}$ are synonymous, then we construct a set $S=\{M_{i},M_{j}\}$ to form a set field. For example, $S_{1}=\{M_{1},M_{2}\}$ and $S_{2}=\{M_{3},M_{4},M_{5}\}$ are two set fields.
- Second, if the entity mentions $M_{i}\in S_{1}$ and $M_{s}\in S_{2}$ are the nearest neighbors or co-occurring in the entity-layer field network, then we construct a set-layer field network $f(S_{1}\;-\;S_{2})$ and consider $S_{1}$ and $S_{2}$ to be related. For example, the network of $S_{1}$ and $S_{2}$ is $f(S_{1}-S_{2})=\{M_{1},M_{2},M_{3},M_{4},M_{5}\}$.

The set-layer field network implicitly provides contextual information at the set layer, ensuring that the generated synonyms are intricately connected to their preceding and following texts, thereby enhancing the coherence and relevance of synonym set generation.

#### Building sentence-layer field network.

We employ the BM25 [26] information retrieval algorithm to evaluate the match degree between sentences and entity mentions. Additionally, we consider factors such as entity mention frequency, sentence length, and sentence frequency to determine the contextual information of the sentence-layer field network $f(T_{1}-T_{2})$.

Specifically, firstly, the entity mention frequency (Term Frequency $TF_{i}$) of the sentence is calculated. $TF_{i}$ indicates the significance of the entity mention in the context of the sentence. Secondly, the inverse sentence frequency ($ICF_{i}$) of the entity mention is calculated. $ICF_{i}$ denotes the rarity of the entity mention item, i.e., its importance in the whole set of sentences. Thirdly, the BM25 Score is calculated based on the $TF_{i}$, $ICF_{i}$, and other parameters of the entity mentions. The formula for the calculation of the BM25 Score is as follows:

$$BM25=I\text{C}F\cdot \frac{TF\cdot (K_{1}+1)}{TF+K_{1}\cdot (1-b+b\cdot \frac{\text{sen\_length}}{\text{avg\_sen\_length}})}$$ (1)

where *ICF* indicates the inverse sentence frequency of the entity mention term. *TF* indicates the word frequency of the entity mention term in the sentence. $K_{1}$ and *b* are the moderation parameters. sen_length indicates the length of the sentence. avg_sen_length indicates the average sentence length. Finally, ranking is executed by sorting the text sentences based on the BM25 score. The sentences with higher scores are placed higher in the results. The *top*-*k* sentences with the highest scores are filtered out based on the sentence-layer field network.

**Fig 5:**
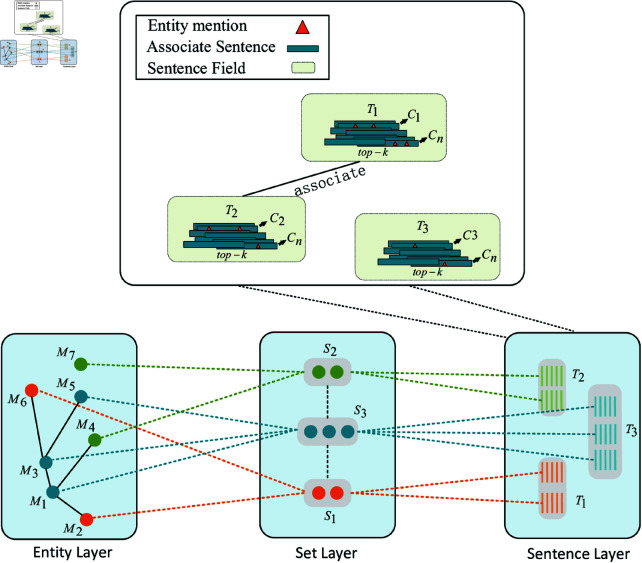
Sentence-layer field network.

We construct the sentence-layer field network to capture sentence-layer contextual information. As illustrated in Fig 5, the sentence-layer field network consists of numerous candidate sentences. To capture sentence-to-sentence semantic relationships, we construct the sentence-to-sentence associate semantic field network $f(T_{1}-T_{2})$ using the following procedures:

- First, we use the BM25 information retrieval algorithm to obtain the *top*-*k* sentences with the highest correlation as the sentence fields of the entities.
- Second, if the entity mentions $M_{i}\in C_{1}$ (where $C_{1}\in T_{1}$) and $M_{s}\in C_{s}$ (where $C_{2}\in T_{2}$) are associated in the set-layer field network, then we construct a sentence-layer field network $f(T_{1}-T_{2})=\{C_{1},C_{2},\ldots ,C_{n}\}$ and consider that $T_{1}$ and $T_{2}$ are connected.

The sentence-layer field network employing the BM25 algorithm effectively filters and ranks candidate sentences. This ensures that the chosen contextual information is highly pertinent to the entity’s context, offering a more precise reflection of the entity’s usage and meaning in diverse contexts. Consequently, the generated synonym set becomes enriched with semantic details, enhancing its practical applicability and accuracy.

### Flexible perceptual field neural network classifier

**Fig 6:**
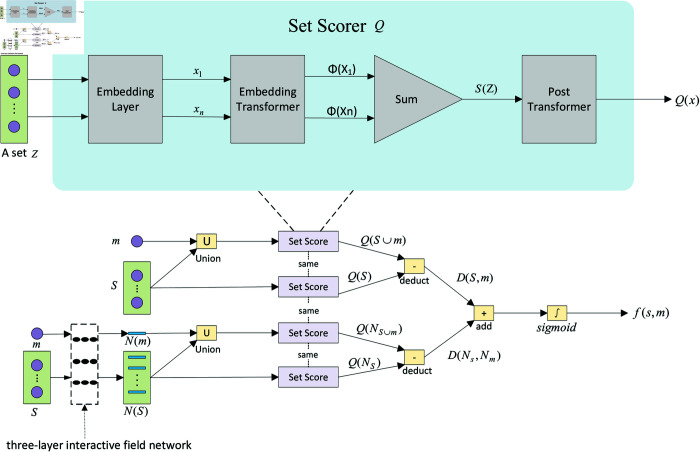
The architecture of the flexible perceptual field neural network classifier.

As shown in Fig 6, following data preparation and the construction of a three-layer interactive field network, we construct a flexible perceptual field neural classifier, denoted as *f*(*S*,*m*), which determines whether the set of synonyms *s* should contain candidate entity *m*.

#### Flexible perceptual field neural network classifier architecture.

The fundamental objective of the flexible perceptual field neural network classifier is to assess whether a candidate entity *m* is suitable for inclusion in the synonym set *s*.

A previous study [27] demonstrated that the crux of learning synonym sets lies in the replacement invariance of the term classifier for synonym sets when new terms are introduced. They initially utilized a set tagger to ascertain the scores of the original synonym set and then determined the scores of the newly formed synonym set (i.e., the set to which new terms are added) using the same set tagger. To translate the disparity between these two scores into a probability, they applied an objective function involving sigmoid units. However, this approach solely utilizes entity embedding features to acquire the synonym signal, neglecting contextual information about entity synonymy between the entity set layer and the sentence layer.

Based on the three-layer interaction field network, this paper builds a flexible perceptual field neural classifier to holistically model entities, sets, and sentences. As shown in Fig 6, the lower part of the figure illustrates the overall framework of classifier *f*(*S*,*m*), which mainly includes multiple parallel raters *Score*(*x*). The upper part of the figure shows the specific architecture of each rater *Score*(*x*), which takes set *x* as input, passes it through the scoring system, and ultimately produces a quality score *Q*(*x*). This score *Q*(*x*) reflects the integrity and consistency of set *x*.

As shown in Fig 6, given a synonym set $S=\{s_{1},s_{2},\ldots ,s_{n}\}$, a candidate entity *m*, and a three-layer interaction field network N={f(M−M),f(S−S),f(T−T)}, we construct the flexible perceptual field neural network classifier as the following steps:

- First, the sets $S=\{s_{1},s_{2},\ldots ,s_{n}\}$ and $Sum=\{s_{1},s_{2},\ldots ,s_{n},m\}$ are input to the set scorer to obtain two scores, *Q*(*S*) and *Q*(*Sum*), respectively. The difference between *Q*(*Sum*) and *Q*(*S*) is calculated, denoted as follows:
  D(S,m)=Q(Sum)−Q(S) (2)
- where *Q*(*S*) is the quality score of the input synonym set *S*, and *Q*(*Sum*) represents the quality score after adding the candidate entity *m* into the synonym set *S*.
- Second, based on the three-layer interactive field network, we learn the entity representation in a mutually reinforcing way and obtain a flexible perceptual field entity representation. The sets $N(S)=\{s_{1},s_{2},\ldots ,s_{n}\}$ and $N(Sum)=\{s_{1},s_{2},\ldots ,s_{n},m\}$ are input to the set scorer to obtain two scores, $Q(N_{s})$ and $Q(N_{Sum})$, respectively. The difference between $Q(N_{Sum})$ and $Q(N_{s})$ is calculated, denoted as follows:
  $$D(N_{s},N_{m})=Q(N_{Sum})-Q(N_{s})$$ (3)
- where $Q(N_{s})$ is the quality score of the input flexible perceptual field synonym set $N_{s}$, and $Q(N_{Sum})$ is the quality score after adding the flexible perceptual field candidate entity $N_{m}$ to the synonym set $N_{s}$.
- Third, the sum of these two difference scores is computed, and a sigmoid function is applied to transform it into a probability. To determine whether the candidate entity should be added to the synonym set, the probability P(m∈S) is defined as:
  $$P(m\in S)=f(S,m)=sigmoid(D(S,m)+D(N_{s},N_{m}))$$ (4)
- where $sigmoid(x)=\frac{1}{1+e^{-x}}$ is a nonlinear function, *D*(*S*,*m*) is the difference in quality score after adding the candidate entity, and $D(N_{s},N_{m})$ is the difference in quality score after adding the candidate entity to the flexible perceptual field.

We employ log-loss as a loss function to train our classifier *f*(*S*,*m*). ϑ(f) is defined as follows:

ϑ(f)=−log(f(S,m))−log(1−f(S,m))·(1−y) (5)

where if m∈S, then y=1; otherwise, y=0. Next, we describe the quality score architecture Q(·).

#### Scorer architecture.

As shown in Fig 6, following Shen *et al*. [13], the scorer architecture consists of an embedding layer, a transformer, a summation operation, and a post-transformer network. The scoring architecture operates as follows:

- First, given a set of items $Z=\{z_{1},z_{2},\ldots ,z_{n}\}$, the set scorer initially feeds each item $z_{i}$ into an embedding layer to derive its corresponding embedding vector, denoted as $x_{i}$.
- Second, employing the embedding transformer ϕ(·) (a fully connected neural network with two hidden layers), we transform the original embedding vector into a new item representation, $\phi (x_{i})$.
- Third, a summation operation is performed on all the transformed term representations $\phi (x_{i})$ to yield the original set representation:
- Last, set representation *S*(*z*) is fed into a post-hidden layer transformer ϕ(·) (a fully connected neural network with three hidden layers) that produces the ultimate set quality score.

### Dynamic-weight-based set generation algorithm

In the preceding study [27], a set generation algorithm was introduced, leveraging a set scorer to generate synonym sets from candidate entities $v_{i}\in V$. The algorithm employed the set scorer to compute a probability *P* to determine whether a new candidate entity $v_{i}$ should be added to the synonym set $S_{i}$. If *P* is greater than the threshold *a*, then the entity $v_{i}$ is added to the set $S_{i}$; otherwise, a new set is created and added to the pool of sets denoted as Pool.

However, the study [28] exclusively relies on entity embedding features to capture synonym signals, neglecting the contextual information of entity synonyms between the set layer and the sentence layer. Furthermore, with an increasing number of sets, the set generation algorithm may face an issue of imbalanced set generation, wherein the model tends to exhibit bias toward larger sets. This issue could potentially result in a reduction in accuracy during synonym set generation.

To deal with the above issue, we design a dynamic-weight-based set generation algorithm. The algorithm solves the issue of imbalance in set generation and improves its accuracy. For sets with fewer synonyms, dynamic weights can increase their influence in training, making the model focus more on important features that might be obscured by lower-frequency sets.

To address the challenge of set imbalance, the dynamic-weight-based set generation algorithm employs a data sampling technique and introduces a weighted cross-entropy function. The algorithm aims to rebalance the distribution of samples across different sets by adjusting set weights. By assigning distinct weights to different sets, the function is tailored to guide the model toward effectively discerning sets with fewer entities. The algorithm proposes calculating the weight for each set based on the number of entities in different synonym sets. Specifically, it utilizes the inverse class frequency as weights, i.e., computing the total number of entities divided by the number of entities in each set. The algorithm fine-tunes the model by assigning higher weights to sets with fewer instances, which mitigates the effect of set imbalance. Details are provided below:

$$ICF=\log\left(\frac{H}{S_{c}+\epsilon }+1\right)$$ (6)

where *H* is the total number of entities, $S_{c}$ is the number of synonymous entities belonging to category set *S*, and ϵ is a small constant added to prevent division by zero.

The set weights are then considered using a weighted cross-entropy function, which multiplies the cross-entropy of each set by its corresponding weight and averages over all set samples. Details are given below:

$$L=-\frac{1}{H}\sum_{i=1}^{H}(ICF_{i}\cdot Y_{i}\cdot \log(P_{i}))$$ (7)

where $ICF_{i}$ represents the weight of the set to which the *i^th^* entity belongs, $Y_{i}$ stands for the true label, and $P_{i}$ denotes the predicted probability.

This dynamic weight assignment aims to ameliorate the issue of imbalance by automatically adjusting the weights of various sets during each training iteration. As a result, the candidate entities in each iteration are predominantly compared with synonym sets bearing larger weights. In this research, we directly set the probability threshold *a* as 0.5 and explore its impact on clustering performance in subsequent analyses.

**Algorithm 1 Dynamic-weight-based set generation algorithm**.

**Require** (1) flexible perceptual field neural network classifier

  *f*(*S*,*m*); (2) vocabulary of candidate entities $V=\{v_{1},v_{2},\ldots ,v_{n}\}$;

  (3) probability threshold a∈(0,1).

**Ensure** Entity synonym set pool $S=\{S_{1},S_{2},\ldots ,S_{n}\}$, where $S_{i}\subseteq V$,

  ${⋃}_{i=1}^{m}S_{i}=V$, and $S_{i}\cap S_{j}=\emptyset$ for i≠j

  Create and add the first cluster, $S_{1}$, to Entity Synonym Set

  Pool

  **for** each candidate entity $v_{i}$ in *V* **do**

   best_score ← 0;

   best_j←1;

   **for** each set $S_{j}$ in Entity Synonym Set Pool **do**

    total_entities ← sum of entities in Synonym Set Pool;

    weight($S_{j}$) ← total_entities / (number_of_entities_in_set$\left(S_{j}\right)$ +1);

    $S_{min\_weight}$ ← min(sets, key=get_set_weight);

    **if** $f(S_{min\_weight},v_{i})>$ best_score **then**

     best_score $\leftarrow f(S_{min\_weight},v_{i})$;

     best_j ←j;

    **end if**

    **if** best_score >*a* **then**

     $S_{best\_j}$.add($v_{i}$);

    **else**

     *S*.append($\left\{v_{i}\right\}$);

    **end if**

   **end for**

  **end for**

  return *S*

As shown in Algorithm 1, the input of the algorithm comprises three parts: (1) a flexible perceptual field neural network classifier *f*(*S*,*m*), (2) a vocabulary list of candidate entities $V=\{v_{1},v_{2},\ldots ,v_{n}\}$, and (3) a probability threshold *a*. The output of the algorithm is an entity synonym set pool $S=\{S_{1},S_{2},\ldots ,S_{n}\}$ clustered from the vocabulary *V*.

Specifically, the algorithm enumerates each $v_{i}\in V$ once and maintains a pool containing all identified sets $S_{i}$. Within the pool, dynamic weight allocation is applied to adjust the weight of each set dynamically. The candidate words entered in each iteration are preferentially compared with sets of lower weight. For candidate entities $v_{i}$, we apply the flexible perceptual field neural network classifier to compute the probability of adding this entity into each identified set in *S*.

If this probability exceeds the threshold *a*, $v_{i}$ is added to the set $S_{i}$. Otherwise, if the probability falls below *a*, a new set $\left\{v_{i}\right\}$ is created using this candidate entity and added to the set pool. This iterative process continues until the entire vocabulary has been traversed once. The algorithm then returns all the detected synonym sets.

## Experiments

In this section, to evaluate the effectiveness of the EnSynFields approach in generating synonym sets, we perform experiments on three real-world public datasets. Initially, we outline the experimental setup, followed by the presentation of experimental results. Finally, we delve into a comprehensive analysis of each component of EnSynFields, showcasing several specific case studies.

### Experimental design

We commence by delineating the dataset, the comparative approaches, and the model variables. Additionally, we outline the experimental setup proposed for EnSynFields.

#### Datasets.

We evaluated EnSynFields on three public benchmark datasets of real-world.

**Wiki.** Wiki contains approximately 100,000 articles extracted from Wikipedia, comprising a total of 6,839,331 sentences. The Freebase knowledge base (https://developers.google.com/freebase?hl=zh-cn) is utilized to enrich and annotate the Wiki dataset.

**NYT.** NYT consists of 118,664 news articles from *The New York Times* (2013), totaling 3,002,123 sentences. The Freebase knowledge base is employed to generate NYT dataset.

**PubMed.** PubMed includes approximately 1.5 million abstracts of research papers from PubMed (https://www.ncbi.nlm.nih.gov/pubmed), amounting to 15,051,203 sentences. The UMLS knowledge base (https://www.nlm.nih.gov/research/umls/) is applied to enhance and structure the PubMed dataset.

DBpedia Spotlight [25] serves as the entity linker for the Wiki and NYT datasets, while PubMed utilizes PubTator [29]. Additionally, knowledge bases such as Freebase [30] and UMLS [31] are employed. For test set creation, a subset of linked entities was randomly selected, with the remaining entities reserved for training. The details of the datasets are summarized in Table 1.

**Table 1:** Used datasets and knowledge bases.

Dataset	Wiki	NYT	PubMed
#Documents	100,000	118,664	1,554,433
#Sentences	6,839,331	3,002,123	15,051,203
#Entity Mentions	98,664	31,702	96,588
#Entity Synonym Sets	4,920	1,494	17,972
#Entity.Fields	98,664	31,702	96,588
#Training Entities	8,731	2,600	72,672
#Training Synonym Sets	4,359	1,273	28,600
#Test Entities	891	389	1,743
#Test Synonym Sets	256	117	250
Knowledge base	Freebase	Freebase	UMLS

#### Approaches of comparison.

**Kmeans.** Kmeans is an unsupervised clustering algorithm [32]. This approach takes candidate entity embeddings as input to the algorithm, and the output is the set of detected synonyms. It is used to cluster synonym sets within the entity vocabulary.

**Louvain.** Louvain is an unsupervised algorithm for discovering the structure of network communities based on modularity optimization [33]. This approach constructs a graph of candidate entities, where each candidate entity represents a node. The embedding of each candidate entity is then calculated using cosine similarity, and an edge is added to the graph if the similarity exceeds a predefined threshold.

**SVM+Louvain.** SVM+Louvain is a two-stage supervised approach [34], where the initial stage uses SVM to predict synonym pairs, and the second stage involves applying the predicted synonym pairs to construct a graph, which is then processed using the Louvain algorithm to obtain a synonym set.

**SetExpan+Louvain.** SetExpan+Louvain is a two-stage supervised approach, where the initial stage uses SetExpan (a weakly supervised set expansion algorithm) [34] to find the *K* nearest neighbors of each candidate entity in a vocabulary list and then constructs a *k*-NN graph. In the second stage, the Louvain algorithm is applied to this graph to obtain the set of synonyms.

**COP-Kmeans.** COP-Kmeans is a semi-supervised variant of Kmeans, combining the COP and Kmeans algorithms for clustering [35]. It utilizes constraint information to guide the clustering process, improving accuracy and stability. The approach sets the oracle number *K* of clusters for each dataset and converts the training synonyms into pairwise constraints.

**SynSetMine.** SynSetMine is a two-stage supervised approach [13], where the initial stage trains a classifier. In the second stage, a set generation algorithm is employed to generate a set of synonyms.

**EnSynFields.** EnSynFields is our proposed approach for generating entity synonym sets based on flexible perceptual fields. It combines multi-layer contextual information with a dynamic-weight-based algorithm to develop synonym sets from the candidate entity lexicon.

#### Hyper-parameter setting.

For entity embedding, we refer to the 50-dimensional entity embedding approach published by Shen *et al*. [13]. To ensure a fair comparison across datasets, we fix the dimensionality of the associative item embedding at 50. To tune the model hyper-parameters, we employ a five-fold cross-validation approach.

For EnSynFields, we use two hidden-layer embedding transformers of sizes 50 and 250 for the embedded feature layer and three post-hidden-layer transformers of sizes 250, 500, and 250. We apply the Adam optimization algorithm with an initial learning rate of 0.001 to optimize the EnSynFields model. Additional model hyper-parameters are provided in Table 2.

**Table 2:** Hyper-parameter settings.

Hyper-parameter name	NYT	WIKI	PubMed
Learning rate	0.001	0.001	0.003
Dropout rate	0.3	0.4	0.3
FieldSize	5	5	5
*a*	0.5	0.5	0.5

#### Evaluation metrics.

Based on previous research work, we adopted three standard clustering metrics that were used as evaluation metrics for this experiment.

**ARI:** ARI is a prevalent metric for gauging the similarity of two cluster assignments. A given model predicts a cluster assignment denoted by *C*^1^, and the true cluster assignment is denoted by *C*^2^. ARI is calculated as follows:

$$ARI=\frac{RI-E(RI)}{\max(RI)-E(RI)}$$ (8)

$$RI=\frac{TP+TN}{TP+TN+FP+FN}$$ (9)

where *TP* denotes the number of element pairs assigned to the same cluster in both *C*^1^ and *C*^2^, while *TN* represents the number of element pairs assigned to different clusters in both *C*^1^ and *C*^2^. *FP* denotes the number of element pairs assigned to the same cluster in *C*^1^ but not in *C*^2^, and *FN* represents the number of element pairs assigned to the same cluster in *C*^2^ but not in *C*^1^. The total number of element pairs is given by TP+TN+FP+FN.

**FMI:** FMI is another similarity measure that evaluates the agreement between two cluster assignments, using precision and recall to calculate similarity. Details are as follows:

$$FMI=\frac{TP}{\sqrt{\left(TP+FP)(TP+FN\right)}}$$ (10)

where *TP* denotes the number of element pairs correctly assigned to the same cluster in both *C*^1^ and *C*^2^, *FP* represents the number of element pairs assigned to the same cluster in *C*^1^ but not in *C*^2^, and *FN* denotes the number of element pairs assigned to the same cluster in *C*^2^ but not in *C*^1^.

**NMI:** NMI computes normalized mutual information between two cluster distributions, which consists of mutual information (MI) and information entropy (IE) as shown below:

$$NMI(X,Y)=\frac{I(X,Y)}{\sqrt{H(X)H(Y)}}$$ (11)

where *I*(*X*,*Y*) is the mutual information (MI) between *X* and *Y*, and *H*(*X*) and *H*(*Y*) are the information entropy (IE) of *X* and *Y*, respectively.

### Experimental results

In this study, we conduct experiments to evaluate the generation of entity synonym sets in terms of both efficiency and effectiveness. To fully understand the experimental results, this section is divided into two main parts: clustering performance and flexible perceptual field neural network classifier prediction performance.

#### Clustering performance.

Table 3 presents a comparison of clustering performance, where the best results are underlined. From the results, we observe that EnSynFields outperforms the baseline approaches across three publicly available datasets.

**Table 3:** Performance comparison of entity synonym set generation.

Approach	NYT			Wiki			PubMed		
	ARI(%)	FMI(%)	NMI(%)	ARI(%)	FMI(%)	NMI(%)	ARI(%)	FMI(%)	NMI(%)
Kmeans	28.9	30.9	83.7	34.4	35.5	87	48.7	49.9	88
Louvain	21.8	30.6	90.1	42.3	46.5	92.6	46.6	52.8	90.5
SVM+Louvain	3.6	5.1	21	6	7.6	25.4	7.8	8.8	31.1
SetExpan+Louvain	43.9	44.3	90.3	44.8	45	92.1	58.9	61.9	92.2
COP-Kmeans	33.8	34.6	87.9	38.8	40	90.3	49.1	51.9	89.9
SynSetMine	44.9	46.4	90.6	56.4	57.1	93	74.3	74.5	95
EnSynFields	48.6	50.0	91.5	58.7	60.5	93.8	77.6	78.2	96.3

As shown in Table 3, the unsupervised approaches Kmeans and Louvain exhibit lower performance than EnSynFields, mainly because they cannot leverage labeled signals, which limits their effectiveness when such labels are available. SetExpan+Louvain demonstrates lower performance compared to EnSynFields but significantly outperforms Kmeans and Louvain. This is because SetExpan+Louvain is a two-stage supervised algorithm that utilizes *K*-nearest neighbor information along with Louvain’s algorithm.

In contrast to the Kmeans algorithm, COP-Kmeans integrates additional supervised data from the training set, which enhances its performance through more informed clustering decisions. We observe that SVM+Louvain performs significantly worse than the other approaches, suggesting that using SVM to capture supervised information results in poor performance for synonym set generation. The primary drawback of SVM+Louvain stems from the fact that its learning model lacks a holistic view of the set and relies solely on pairwise similarity.

SynSetMine outperforms other approaches in terms of performance but still falls short of EnSynFields. This is because SynSetMine only considers semantic relations at the entity layer, ignoring the broader global entity context, which could substantially improve the quality of synonym set discovery. The above analysis suggests that the flexible perceptual field neural network classifier and the dynamic-weight-based set generation algorithm enhance the efficiency of entity synonym set expansion.

#### Flexible perceptual field neural network classifier performance.

**Fig 7:**
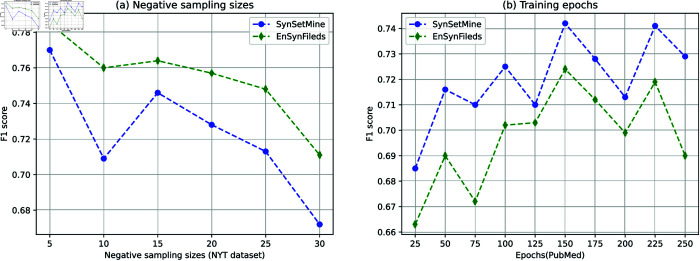
Comparing F1 scores across different negative sampling sizes and epochs.

To evaluate neural network classifiers with flexible perceptual fields, the F1 scores of EnSynFields and SynSetMine are compared using datasets from NYT and PubMed. Fig 7(a) shows these scores for varying negative sample sizes based on the NYT dataset. Observations reveal that EnSynFields consistently achieves a higher F1 score compared to SynSetMine. As depicted in Fig 7(b), this trend is evident across various training periods on the PubMed dataset, with EnSynFields maintaining superior F1 scores throughout.

The above results show that the flexible perceptual field neural network classifier captures entities, sets, and sentences as a whole, and integrates them into the dynamic-weight-based set generation algorithm. This integration effectively enhances the model’s predictive ability for synonym sets.

#### Semantic similarity evaluation.

To further evaluate the semantic consistency of the generated synonym sets, we incorporate BERT-based cosine similarity as an additional metric. Unlike clustering metrics, which evaluate structural accuracy, BERT similarity quantifies the semantic coherence of words within the same synonym set.

Table 4 presents the BERT cosine similarity scores across datasets, alongside their corresponding NMI scores for comparison.

**Table 4:** Comparison of BERT cosine similarity and NMI scores across datasets.

Dataset	BERT Cosine Similarity	NMI (%)
NYT	**0.877**	91.5
Wiki	**0.899**	93.8
PubMed	**0.923**	96.3

We observe a positive correlation between BERT similarity and NMI scores across datasets. Specifically, PubMed, which exhibits the highest NMI (96.3%), also achieves the highest BERT similarity (0.923), while NYT, with the lowest NMI (91.5%), obtains a relatively lower similarity score of 0.877.

This suggests that our model not only performs well in clustering structure but also preserves strong semantic relationships within synonym sets. However, despite these high similarity scores, some errors still occur, particularly when contextually related but semantically distinct words are grouped together.

### Model analysis

This section examines how various parameters, such as hidden layer sizes, embedding dimension, and threshold *a*, influence performance from multiple perspectives.

#### Analysis of different hidden layer sizes.

**Fig 8:**
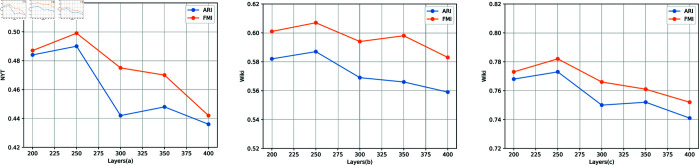
Results of different layer sizes for scorer architecture.

To assess the impact of different hidden layers on the scorer architecture more comprehensively, we use different hidden layer sizes for analysis. For the embedding transformer of scorer architecture, the different hidden layer sizes are denoted as 200, 250, 300, 350, 400. For the post-hidden layer transformer of scorer architecture, the different hidden layer sizes are denoted as 200 *2, 250*2, 300*2, 350*2, 400*2. Fig 8 shows the ARI and FMI for the different scorer architecture across the three datasets. It can be observed that the performance improvement of each architecture is noticeable when the hidden layer size transitions from 200 to 250. However, the performance of each architecture generally declines as the hidden layer increases. Therefore, we consider that embedding transformer hidden layer sizes ranging from 200 to 250, and post-hidden layer transformer sizes ranging from 400 to 500, are adequate for capturing the essential synonymous signals required for identifying entity synonymous relationships.

#### Effect of dimension of embeddings.

**Fig 9:**
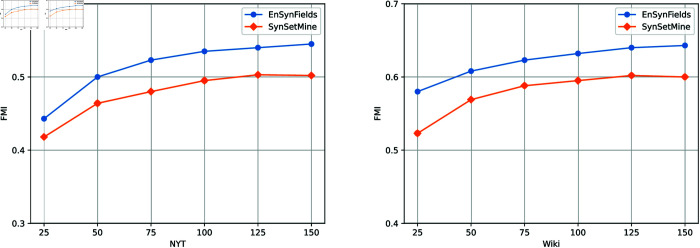
Comparing F1 scores across different negative sampling sizes and epochs.

In representation learning, the size of embeddings significantly impacts the efficacy of machine learning models. We explored this by fixing all other hyper-parameters and varying the embedding dimension across 25, 50, 75, 100, 125, 150. Fig 9 illustrates the influence of embedding size on the performance of SynSetMine and EnSynFields using NYT and Wiki datasets.

We observe that EnSynFields consistently outperforms SynSetMine, and if the dimensionality is not large enough, the model suffers from underfitting, leading to convergence difficulties. For example, the model performs poorly when the embedding dimension is set to 25. Performance improves gradually when the dimension exceeds 50. Moreover, the experimental results indicate that our model remains stable across varying embedding sizes.

#### Effect of different probability threshold *a.*

Algorithm 1 outlines our set generation approach, which uses a probability threshold to decide the inclusion of a candidate entity in existing sets. A higher threshold implies a more conservative approach by the algorithm, leading to the creation of additional sets.

**Fig 10:**
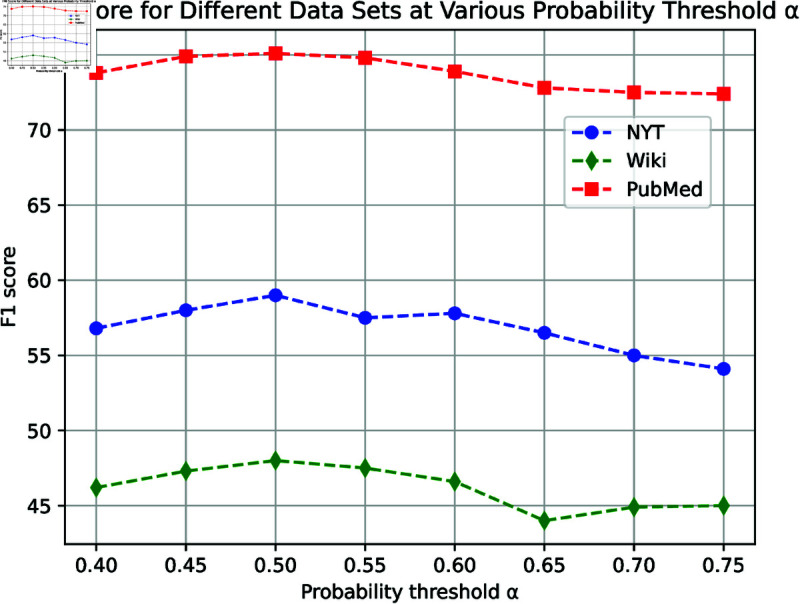
Results of different probability thresholds *a.*

This experiment focuses on examining the impact of this hyper-parameter on the experimental outcomes. We execute the set generation algorithm using a flexible perceptual field neural network classifier and vary the threshold to observe its effects. Fig 10 displays the results.

Initially, we observe that clustering effectiveness remains stable across different thresholds, with values between 0.4 and 0.6 often being more suitable. Furthermore, a threshold of 0.5 emerges as a robust choice, consistently yielding favorable outcomes across different datasets.

### Case studies

Table 5 presents example outputs generated by EnSynFields. The output of entity synonym sets from our approach is randomly selected. We observed that our approach can generate entity synonym sets of different types and domains. For example, the prediction results of “film” and “movie” are the correct synonym, and “infant” and “baby” are also the correct synonym. However, our approach is not perfect and sometimes produces incorrect results. For instance, the prediction of “purse” and “medical doctor” as synonyms is incorrect. This failure case suggests that our model may struggle with distinguishing between contextually similar but semantically different words. Future improvements could involve integrating more fine-grained contextual embeddings to mitigate such errors.

**Table 5:** Entity synonym set output examples (G denotes the ground truth, and O denotes the output results of our approach).

EnSynFields					
Output	O	G	Output	O	G
{Britain,UK,united_kingdom}	1	1	{teenagers,adolescence}	1	1
{biking,bicycling,cycling}	1	1	{infant,baby,newborn}	1	1
{fabric,textile,cloth}	1	1	{purse,handbag,medical_doctor}	1	0
{film,movie,cinema}	1	1	{mother,mom,mum}	1	1
{rocks,rock,stone}	1	1	{gas,gasoline,petrol}	1	1

## Conclusion

This paper presents an approach to extract entity synonym sets from text corpora. We construct a three-layer interaction field network to capture contextual information across the entity layer, set layer, and sentence layer. To determine the inclusion of a candidate entity in a synonym set, we developed a flexible perceptual field neural network classifier and incorporated it into a dynamic weight-based algorithm for new entity synonym set detection.

In the evaluation of our proposed approach, it was implemented across three distinct real-world synonym set datasets and compared with several contemporary state-of-the-art approaches. The empirical findings indicate that our approach exhibits enhanced efficacy in the task of entity synonym set generation, surpassing the performance of the most advanced existing techniques.

In subsequent research endeavors, our focus will be on investigating the potential of leveraging entity attribute information to augment the efficacy of entity synonym generation approaches. Additionally, we plan to design a multimodal strategy aimed at distinguishing near-sense entities from synonymous entities, thereby enhancing the precision of augmented entity synonym sets.
